# Nephroprotective Effect of *Sonchus oleraceus* Extract against Kidney Injury Induced by Ischemia-Reperfusion in Wistar Rats

**DOI:** 10.1155/2018/9572803

**Published:** 2018-02-14

**Authors:** Liliana Torres-González, Eduardo Cienfuegos-Pecina, Marlene M. Perales-Quintana, Gabriela Alarcon-Galvan, Linda E. Muñoz-Espinosa, Edelmiro Pérez-Rodríguez, Paula Cordero-Pérez

**Affiliations:** ^1^Liver Unit, Department of Internal Medicine, University Hospital “Dr. José E. González”, Universidad Autónoma de Nuevo León, Av. Gonzalitos No. 235 Col. Mitras Centro C.P., 64460 Monterrey, NL, Mexico; ^2^Transplant Service, University Hospital “Dr. José E. González”, Universidad Autónoma de Nuevo León, Av. Gonzalitos No. 235 Col. Mitras Centro C.P., 64460 Monterrey, NL, Mexico; ^3^Basic Science Department, School of Medicine, UDEM, Universidad de Monterrey, Av. Ignacio Morones Prieto 4500 Pte., Jesús M. Garza, C.P., 66238 San Pedro Garza García, NL, Mexico

## Abstract

**Introduction:**

Kidney ischemia-reperfusion (I/R) injury is the main cause of delayed graft function in solid organ transplantation. *Sonchus oleraceus* is a plant with well-known antioxidant and anti-inflammatory activities; however, its effects on renal I/R are unknown.

**Objective:**

To evaluate whether *S. oleraceus* extract (S.O.e.) has nephroprotective activity in an I/R model in Wistar rats.

**Materials and Methods:**

Animal groups (*n* = 6): sham, I/R (45 min/15 h), S.O.e (300 mg/kg p.o.), and S.O.e + I/R (300 mg/kg, p.o.; 45 min/15 h). Renal function, proinflammatory cytokines, alanine aminotransferase, markers of oxidative stress, and histology were evaluated.

**Results:**

None of the mediators evaluated differed significantly between the S.O.e and sham groups. Levels of blood urea nitrogen (BUN), creatinine, malondialdehyde (MDA), and proinflammatory cytokines were higher, and superoxide dismutase (SOD) was lower in the I/R group than in the sham group. Histology showed tubular epithelial necrosis in the medulla and cortex in the I/R group. In the S.O.e + I/R group, S.O.e pretreatment attenuated the I/R-induced increases in BUN, creatinine, MDA, and proinflammatory cytokines induced, SOD was maintained, and histology showed discontinuous necrosis in the medulla but no necrosis in the cortex.

**Conclusions:**

S.O.e was neither hepatotoxic nor nephrotoxic. S.O.e. pretreatment showed a nephroprotective effect against I/R.

## 1. Introduction

Kidney injury caused by ischemia-reperfusion (I/R) is a significant clinical problem and is considered to be the main cause of acute renal failure, which can result from shock, partial nephrectomy, or renal transplantation and can lead to morbidity and mortality [1, 2]. Multiple pathogenic factors contribute to the eventual death of kidney cells as a result of I/R, including excessive oxidative stress, actions of proinflammatory cytokines, recruitment of inflammatory cells, and apoptosis [2, 3]. Excessive oxidative stress is caused mainly by reactive oxygen species (ROS) produced during reperfusion and released as part of the inflammatory response. ROS can damage cell organelles and change the oxidation-reduction balance. The action of antioxidants, such as reduced glutathione and enzymes such as superoxide dismutase (SOD), is essential to reducing tissue damage.

Plants have been shown to be potential therapeutic agents to protect against renal I/R. Experimental studies in rats have reported that the primary mechanism through which plant extracts prevent kidney damage induced by I/R involves counteracting the effects of excessive oxidative stress through free radical-scavenging and antioxidant capacity [4, 5]. *Sonchus oleraceus* is a plant of the *Asteraceae* family and is distributed worldwide. Many *Sonchus* species are used by diverse cultures for the treatment of stomach pain, hepatitis, infections, headache, rheumatism, cancer, warts, ulcers, spider and snake bites, and inflammation [6–9]. In Mexico, *S. oleraceus* is known as *Achicoria dulce*, *cerraja*, *lechuguilla*, *muela de caballo*, and *falso diente de león* [10]. Throughout the world, it is known as sow thistle or pūhā [11, 12]. Recent research has shown that an *S. oleraceus* extract (S.O.e) exhibits several types of bioactivity, including anxiolytic [6], anti-inflammatory [7, 9], antibacterial [8], antioxidant [8, 11, 13, 14], antitumor [15], antinociceptive [16], and antiaging [12] activities.

Given the reported antioxidant activity and potential pharmacological properties, the aim of the current investigation was to evaluate whether *S. oleraceus* has a nephroprotective effect against I/R-induced injury in Wistar rats.

## 2. Material and Methods

### 2.1. Extract Preparation

The plant was collected in García, NL, Mexico, during the summer of 2014 and authenticated, and a voucher specimen (UAN-2429) was deposited in the institutional herbarium located at the School of Biology of the Universidad Autónoma de Nuevo León. The *S. oleraceus* aerial part was dried at room temperature for 2 weeks and then finely ground. The extract was obtained using Soxhlet extraction. Briefly, 100 g of dried material was extracted in 1 L of ethanol for 5 h, and the extracted material was filtered and concentrated under reduced pressure at 37°C, dried in an oxygen-free environment at 37°C, and stored at 4°C until use; the recovery was 3.3%.

### 2.2. Animals

Animal procedures were performed in accordance with the proper use and care of laboratory animals and according to the specifications of the Mexican Official Norm NOM-062-ZOO-1999 and were approved by the ethics committee of our institution (HI17-00002). Experiments were performed using male Wistar rats weighing 200–300 g (Círculo A.D.N. S.A. de C.V., Mexico City, Mexico). Animals were kept under standard conditions such as a stable room temperature (24 ± 3°C) and 12 h light-dark cycle and had access to commercial rat pellets and water ad libitum.

### 2.3. Experimental Design

To evaluate whether treatment with S.O.e at a dose of 300 mg/kg could reduce kidney injury after I/R, the following experimental groups were evaluated (*n* = 6 per group).

The sham group was treated with the extract vehicle (3% Tween-20) for 7 days, after which the rats received a sham laparotomy without affecting the renal pedicle.

The S.O.e group was treated with 300 mg/kg of extract for 7 days according to the report by Li et al. [9], after which the rats received the same surgical procedure as the sham group.

The I/R group was treated with the extract vehicle for 7 days, after which acute kidney injury was induced by I/R (45 min ischemia + 15 h reperfusion).

The S.O.e + I/R group was treated with 300 mg/kg of extract for 7 days, after which the kidney injury was induced by the same procedure as the I/R group.

### 2.4. Induction of Kidney Injury

Rats were anesthetized using xylazine (Sedaject; Vedilab S.A. de C.V. Reg. SAGARPA Q-0088-122) by intraperitoneal injection at a dose of 10 mg/kg of body weight with ketamine as an analgesic (Anesket; PiSA Agropecuaria, S.A. de C.V. Reg. SAGARPA Q7833-028) by intraperitoneal injection at a dose of 100 mg/kg of body weight according to the suppliers' specifications.

The I/R and S.O.e + I/R groups received a laparotomy to expose both kidneys. Kidney injury was induced by ischemia caused by 45 min of occlusion of the renal pedicle used vascular clamps, after which the clamps were withdrawn and reperfusion was allowed for 15 h. During this period, rats were allowed access to food and water ad libitum. For the sham and S.O.e groups, the surgical procedure involved a laparotomy without any occlusion.

Blood samples were taken from rats after the surgical procedure and centrifuged at 3500 rpm for 15 min. The serum was separated and was used to measure the levels of alanine aminotransferase (ALT), renal function markers, and proinflammatory cytokines. Kidney tissue samples were obtained immediately after the blood samples were taken. One part of the tissue was fixed in 10% formaldehyde for histopathological evaluation, and the other was frozen at −80°C for measurement of malondialdehyde (MDA) and SOD levels.

### 2.5. Biochemical Analysis, Proinflammatory and Oxidative Stress Markers

Blood urea nitrogen (BUN), creatinine concentration, and ALT activity were determined by spectrophotometry (ILab Aries; Instrumentation Laboratory, Milan, Italy) using commercial kits (Instrumentation Laboratory) according to the supplier's specifications.

The concentrations of proinflammatory cytokines were measured using a commercial enzyme-linked immunosorbent assay for rat interleukin 6 (IL-6), interleukin 1-beta (IL-1*β*), and tumor necrosis factor-alpha (TNF-*α*) (PeproTech, Mexico City, Mexico). Avidin-horseradish peroxidase conjugate was used to oxidize 2,2′-azino-bis(3-ethylbenzothiazoline-6-sulfonic acid), which produced a chromogen whose concentration was proportional to the concentration of the cytokine being evaluated. The measurements were made spectrophotometry at 405 nm.

The concentration of MDA, the final product of lipid peroxidation, was measured using a thiobarbituric acid-reactive substance (TBARS) assay using a TBARS Assay Kit (Cayman Chemical Company, Ann Arbor, MI, USA). To measure MDA concentration, 100 *μ*L of the supernatant from medium or standard, 100 *μ*L of sodium dodecyl sulfate, and 4 mL of the color reagent were added to each vial. The vial was heated at 100°C for 1 h and then immediately cooled in an ice bath and centrifuged at 11,000 rpm for 15 min at 4°C. Next, 150 *μ*L from each vial was transferred to each well in a microplate. The absorbance of the product was measured at a wavelength of 540 nm on a microplate reader. The extent of lipid peroxidation was quantified by estimating the MDA concentration. The results are expressed as micromoles of MDA equivalents formed per liter.

SOD activity was measured using a Superoxide Dismutase Assay kit (Cayman Chemical Company) and a colorimetric assay to measure the concentration of formazan crystals at 450 nm. This assay uses a tetrazolium salt for the detection of superoxide radicals generated by xanthine oxidase and hypoxanthine. To measure SOD activity, 200 *μ*L of the diluted radical detector and 10 *μ*L of the supernatant of tissue homogenate or standard were added to each well of a 96-well plate, and 20 *μ*L of xanthine oxidase was added. Absorbance in the well was measured at a wavelength of 460 nm after 20 min on a microplate reader (Thermo Scientific Multiskan FC, Waltham, USA). The results are expressed as IU/mL. One IU of SOD is defined as the amount of enzyme needed to exhibit 50% dismutation of the superoxide radical.

### 2.6. Evaluation of Renal Histopathology

Kidneys were fixed in a 10% buffered formaldehyde solution (pH 7.4). A representative sample was taken from both kidneys of all rats in the four groups. The tissue was processed routinely and paraffin embedded. Paraffin blocks were cut using a microtome at a thickness of 4 *μ*m, and the sections were deparaffinized, hydrated, and stained with hematoxylin and eosin (H&E). The sections were examined under a microscope for the presence of indicators of cellular damage such as tubular necrosis and eosinophilic casts, which are regarded as semiquantitative measures. A scoring system was used to evaluate kidney histopathology as follows: no damage = 0; mild damage = 1 (unicellular patchy isolated damage); moderate damage = 2 (damage <25%); severe = 3 (damage 25–50%); severe = 4 (>50% damage) [17].

### 2.7. Statistical Analysis

The data are expressed as mean ± standard deviation (SD) and were analyzed by one-way analysis of variance followed by the Tukey test for multiple comparisons or Kruskal-Wallis nonparametric test using Prism software (v. 6.0; GraphPad, San Diego, CA, USA). Differences between means were considered significant at *p* < 0.05.

## 3. Results

### 3.1. Study of the Toxicity of the Plant Extract In Vivo

**Figure 1:**
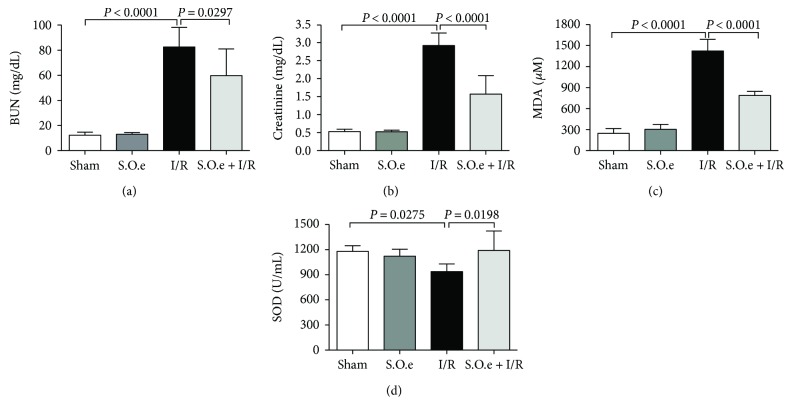
Changes in renal function tests and markers of oxidative stress. (a) BUN, (b) creatinine, (c) MDA, and (d) SOD levels before and after I/R. Values are expressed as mean ± SD.

There were no significant differences between the sham and S.O.e groups in the levels or activities of ALT (86 ± 7 IU/L versus 90 ± 6 IU/L), BUN (12 ± 2 mg/dL versus 13 ± 1 mg/dL), creatinine serum (0.53 ± 0.33 mg/dL versus 0.50 ± 0.22 mg/dL), MDA (247 ± 20 *μ*M versus 304 ± 21 *μ*M), SOD (1082 ± 54 U/mL versus 1179 ± 28 U/mL), and IL-6 (Figure 1, Table 1). The concentrations of IL-1*β* and TNF-*α* were significantly higher in the sham group than in the S.O.e group (Table 1).

### 3.2. Effect of *S. oleraceus* Extract on Kidney Injury Induced by I/R

The BUN and creatinine levels were significantly higher in the I/R group than in the sham group (83 ± 16 mg/dL versus 12 ± 2 mg/dL; 2.92 ± 0.35 mg/dL versus 0.53 ± 0.33 mg/dL, respectively; *p* < 0.0001) (Figure 1). In the group pretreated with *S. oleraceus* before ischemia (S.O.e + I/R group), the increases in BUN and serum creatinine levels were significantly attenuated: 83 ± 16 mg/dL versus 60 ± 21 mg/dL, respectively (*p* = 0.0297) and 2.92 ± 0.35 mg/dL versus 1.57 ± 0.51 mg/dL, respectively (*p* < 0.0001) (Figure 1).

MDA level was significantly higher in the I/R group than in the sham group (1422 ± 166 *μ*M versus 247 ± 20 *μ*M; *p* < 0.0001). *S. oleraceus* treatment significantly attenuated the increase in MDA level in the S.O.e + I/R group compared with the I/R group (789 ± 58 *μ*M versus 1422 ± 166 *μ*M; *p* < 0.001) (Figure 1).

SOD level was significantly lower in the I/R group than in the sham group (959 ± 88 U/mL versus 1082 ± 54 U/mL; *p* = 0.0275). In the S.O.e + I/R group, SOD levels were preserved to compared at I/R group (1191 ± 231 U/mL; *p* = 0.0198) (Figure 1).

The TNF-*α*, IL-1*β*, and IL-6 concentrations were significantly higher in the I/R group than in the sham group (*p* < 0.004). The cytokine concentrations were significantly lower in the S.O.e + I/R group than in the I/R group (*p* < 0.007) (Table 1).

### 3.3. Evaluation of Renal Histopathology

**Figure 2:**
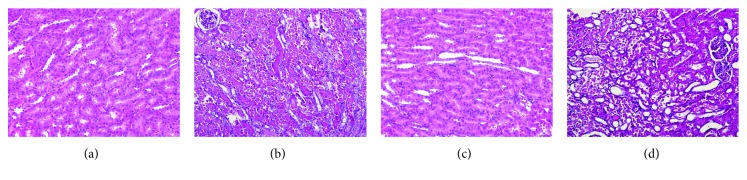
Hematoxylin and eosin (H&E) staining of kidney tissue. (a) Sham group, normal kidney tissue structure (×10); (b) I/R group, diffuse tubular necrosis and abundant protein-like intratubular eosinophilic cylinders (×10); (c) S.O.e group, sham-like morphology; (d) S.O.e + IR group, significant increase in renal histopathological findings (×10).

The scoring system used for evaluation of kidney histopathology is shown in Table 2. The mean score for tissue damage was significantly higher in the I/R group than in the sham group. Kidneys of rats treated with *S. oleraceus* exhibited less damage, as shown by lower scores, compared with the I/R group. Light microscopic examination of H&E-stained tissue sections showed normal renal parenchyma, tubules, and glomeruli in the sham and S.O.e groups. By contrast, kidney tissues from the I/R group showed tubular epithelium necrosis in the medulla and cortex. Kidney tissues from the S.O.e + I/R group showed discontinuous necrosis in the marrow and conserved cortex (Figure 2).

## 4. Discussion

When an extract is proposed as a possible therapeutic strategy, it is necessary to demonstrate that it is not toxic. To rule out any possible hepatotoxic or nephrotoxic effects of the extract, we measured the levels of ALT, BUN, creatinine, SOD, and MDA in the S.O.e group. The extract had no measurable effects on these variables, as shown by the similar levels of these mediators in the treated and sham groups. This agrees with the findings by other investigations that *S. oleraceus* has no cytotoxic effect *in vitro* or *in vivo* [9, 18].

Renal ischemia cannot be prevented in some clinical situations such as renal transplantation, traumatic shock, sepsis, postpartum hemorrhage, or major surgery. I/R-induced kidney injury occurs when the blood flow to an organ is stopped temporarily and blood is then reperfused with oxygenated blood to the organ. This process triggers the release of ROS, one of the main causes of the tissue injury caused by reperfusion [2, 19, 20]. This tissue injury stimulates an inflammatory response mediated mainly by neutrophils and macrophages, which then release proinflammatory mediators such as cytokines [1]. This inflammatory response directly affects renal function as shown by an increase in the serum concentrations of BUN and creatinine. Some plants with antioxidant activity have been reported to decrease the secondary damage caused by oxidative stress produced by kidney I/R (which affects kidney function) [21, 22]. In this study, we found that the pretreatment with S.O.e. attenuated the increases in serum of BUN and creatinine levels compared with those observed in the I/R group. This finding suggests that S.O.e. may have a nephroprotective effect, which may be related to its antioxidant activity as reported by others [8, 11, 13, 14, 18, 23].

Proinflammatory cytokines participate in an important pathway involved in I/R-induced renal injury. IL-6 release increases the degree of injury, dysfunction, and renal inflammation; this cytokine promotes the expression of adhesion molecules and consequent oxidative stress [24]. TNF-*α*, a potent proinflammatory cytokine, has been shown to reduce glomerular perfusion by inducing the synthesis of vasoconstrictor and vasodilator mediators [25]. IL-1*β* has been described as a chemoattractant that recruits leukocytes to the areas of renal inflammation, which leads eventually to kidney damage [26]. Treatment with an extract of *S. oleraceus* has been reported to have an anti-inflammatory effect in several models. In a recent *in vitro* model of the RAW 264.7 mouse macrophage cell line, an extract of *S. oleraceus* had an anti-inflammatory effect after stimulation with lipopolysaccharide, as shown by significant decreases in the levels of IL-1*β*, IL-6, and TNF-*α*. This study also reported a decreased inflammatory response to *in vivo* xylene-induced edema with an extract dose of 300 mg/kg [9]. In our study, the significantly higher IL-1*β*, IL-6, and TNF-*α* levels in the I/R group than in the sham group indicated the presence of an inflammatory response. Pretreatment with S.O.e. at a dose of 300 mg/kg significantly attenuated this increase in IL-1*β*, IL-6, and TNF-*α* levels in the S.O.e + I/R group through effects on the inflammatory response.

Recent phytochemical investigations of *S. oleraceus* have identified secondary metabolites such as sesquiterpene lactones, taraxasterol, luteolin, apigenin, caftaric acid, chicoric acid, villosol, ferulic acid, *β*-sitosterol, ursolic acid, rutin, *β*-daucosterin, and others [9, 13, 27]. Of these secondary metabolites, several bioactive molecules may be involved in vasoprotection from the endothelial injury induced by I/R. Examples include rutin, which exhibits anti-inflammatory activity because of its free radical-scavenging and antioxidant capacities [28]; *β*-sitosterol, which significantly inhibits the TNF-*α*-induced expression of adhesion molecules that play key roles in the inflammatory process and interfere with multiple signaling pathways including cell cycle, apoptosis, proliferation, and metastasis [29, 30]; apigenin, which attenuates the inhibition of vasorelaxation induced by pyrogallol [31] and exerts anti-inflammatory activity by modulating nuclear factor *κ*B activity, reducing inflammatory cytokine production and limiting neutrophil migration toward the inflammatory microenvironment [32]; and luteolin, a potent inhibitor of inflammation that may also help to ensure endothelial integrity [33]. These actions suggest that the anti-inflammatory effect observed in the S.O.e + I/R group may relate to the presence of some of these molecules described in this plant.

Reperfusion after ischemia increases the release of ROS, which are important effectors of cellular injury [18]. Free radicals participate in the physiopathology of renal I/R injury [2]. Cellular antioxidant enzymes such as SOD block the free radical effect; however, overwhelming of these protective activities by excessive production of free radicals causes lipid peroxidation, whose end product is MDA [34, 35]. In our study, treatment with *S. oleraceus* attenuated the oxidative injury produced by renal I/R, as shown by the increase in SOD activity and decrease in MDA activity. This effect may reflect the antioxidant activities described for some molecules contained in extracts of *S. oleraceus* as rutin, luteolin, and chicoric acid [11, 13].

Several studies have reported characteristic lesions of I/R-induced renal injury [1, 3–5, 19–21]. In our study, ischemia for 45 min and reperfusion of 15 h caused damage to renal tissue, as shown by measures of renal dysfunction such as diffuse tubular necrosis and abundant protein-like intratubular eosinophilic cylinders. S.O.e. attenuated the renal injury produced by the I/R, as shown by discontinuous necrosis in the medulla and conserved cortex.

## 5. Conclusion

Our results indicate that S.O.e. was neither hepatotoxic nor nephrotoxic and that pretreatment with S.O.e. markedly attenuated postischemic damage to rat kidneys. Treatment with S.O.e. attenuated the increase in proinflammatory cytokine levels and markers of renal damage and oxidative stress. Our study provides a basis for further identification of which molecules are responsible for this apparent nephroprotective activity of *S. oleraceus*. This is the first report of nephroprotective activity of S.O.e against I/R-induced injury.

## Figures and Tables

**Table 1:** Concentrations of proinflammatory cytokines in the experimental groups.

Experimental groups	IL-6 (ng/mL)	IL-1*β* (ng/mL)	TNF-*α* (ng/mL)
Sham	0.14 ± 0.03	0.86 ± 0.13	0.42 ± 0.07
I/R	0.37 ± 0.06^∗^	1.36 ± 0.09^∗^	0.77 ± 0.15^∗^
S.O.e	0.13 ± 0.05	0.61 ± 0.11^∗^	0.29 ± 0.10^∗^
S.O.e + I/R	0.11 ± 0.05^∗∗^	0.64 ± 0.19^∗∗^	0.42 ± 0.09^∗∗^

Data are presented as mean ± S.D. ^∗^Sham versus study group (*P* < 0.004), ^∗∗^I/R versus S.O.e + I/R (*P* < 0.007).

**Table 2:** Mean scores for total tissue damage in the experimental groups.

Experimental groups	Parameters	
	Tubular necrosis	Eosinophilia casts
Sham	0.50 ± 0.75	0.00 ± 0.00
I/R	4.00 ± 0.00^∗^	2.25 ± 0.95^∗^
S.O.e	0.50 ± 0.54	0.00 ± 0.00
S.O.e + I/R	2.77 ± 0.66^∗∗^	1.44 ± 0.52^∗∗^

Data are presented as mean ± S.D. ^∗^*P* < 0.05 as compared with Sham, ^∗∗^*P* < 0.05 as compared with I/R.

## Abbreviations

I/R: Ischemia/reperfusion
S.O.e.: *Sonchus oleraceus* extract
ROS: Reactive oxygen species
SOD: Superoxide dismutase
MDA: Malondialdehyde
ALT: Alanine aminotransferase
BUN: Blood urea nitrogen
IL-6: Interleukin 6
IL-1*β*: Interleukin 1-beta
TNF-*α*: Tumor necrosis-alpha.
